# Mathematically Improved XGBoost Algorithm for Truck Hoisting Detection in Container Unloading

**DOI:** 10.3390/s24030839

**Published:** 2024-01-27

**Authors:** Nian Wu, Wenshan Hu, Guo-Ping Liu, Zhongcheng Lei

**Affiliations:** 1 School of Electrical Engineering and Automation, Wuhan University, Wuhan 430072, China; nian.wu@whu.edu.cn (N.W.); zhongcheng.lei@whu.edu.cn (Z.L.); 2 Center for Control Science and Technology, Southern University of Science and Technology, Shenzhen 518055, China; liugp@sustech.edu.cn

**Keywords:** truck hoisting detection, non-intrusive measurement, XGBoost model, abnormality detection

## Abstract

Truck hoisting detection constitutes a key focus in port security, for which no optimal resolution has been identified. To address the issues of high costs, susceptibility to weather conditions, and low accuracy in conventional methods for truck hoisting detection, a non-intrusive detection approach is proposed in this paper. The proposed approach utilizes a mathematical model and an extreme gradient boosting (XGBoost) model. Electrical signals, including voltage and current, collected by Hall sensors are processed by the mathematical model, which augments their physical information. Subsequently, the dataset filtered by the mathematical model is used to train the XGBoost model, enabling the XGBoost model to effectively identify abnormal hoists. Improvements were observed in the performance of the XGBoost model as utilized in this paper. Finally, experiments were conducted at several stations. The overall false positive rate did not exceed 0.7% and no false negatives occurred in the experiments. The experimental results demonstrated the excellent performance of the proposed approach, which can reduce the costs and improve the accuracy of detection in container hoisting.

## 1. Introduction

With the rapid growth of international and domestic trade volume, the loading and unloading operations of containers have become increasingly busy [1,2], emphasizing the importance of safety in port operations [3]. One typical issue in these operations is mistakenly hoisting trucks when unloading containers with cranes. The detection of truck hoisting can effectively minimize economic losses in port operations and guarantee operation safety [4], thus improving the efficiency of normal operations. Since trucks for the transportation of containers are mostly equipped with multiple pairs of locks, containers can be fixed to trucks securely by locking four corresponding locks during transportation, preventing the risk of containers tipping over or slipping. The failure to unlock all locks when unloading containers can result in the hoisting of trucks, which can cause economic losses. Moreover, human safety deserves more concern; therefore, many researchers have devoted time to the detection of truck hoisting.

One method for truck hoisting detection utilizes cameras to ascertain separation statuses between containers and trucks. The manual monitoring method requires the gradual elevation of containers, halting at a specific height, which limits the efficiency of unloading. An alternative method (named PSLS) utilizes photoelectric sensors [5,6] and laser scanners [7,8,9]. PSLS identifies abnormal hoists by assessing the presence of obstacles under containers and monitoring dynamic positional changes. Compared to manual monitoring, PSLS presents superior efficiency. However, photoelectric sensors require frequent manual calibrations and testing, while laser scanners involve substantial initial costs and need regular maintenance and testing. Additionally, the effectiveness of PSLS relies on the accuracy of elevation and the detection precision of the sensors [10,11]. Some studies [12,13] have introduced methodologies for the acquisition of on-site images and videos through the use of cameras, followed by the application of algorithms for target detection [14,15] and feature point matching [16,17] to determine the positional changes of trucks. Nevertheless, adverse weather conditions significantly impact the image and video capture capability of cameras, making it challenging to ensure accuracy [18].

In summary, the above-mentioned methods for hoisting detection in trucks mainly depend on laser detectors, position sensors, and cameras [19]. Instruments such as lasers and infrared detectors require frequent manual calibration, resulting in high maintenance costs and being difficult to popularize. Present methods for truck hoisting detection necessitate a period of hovering after containers are hoisted, constraining the efficiency of unloading processes and increasing power consumption. Moreover, some detection systems are relatively expensive, providing minimal benefits to ports and hindering widespread adoption. Devices used in conventional methods, such as lasers and cameras, are also susceptible to on-site disturbances, such as vibrations, fog, and intense light, imposing stringent demands on sensor stability and precision.

To address the above-mentioned issues, an indirect detection approach is proposed in this paper. The proposed approach involves the collection of electrical signals, including voltage and current, and the employment of mathematical and XGBoost models [20,21] to discern instances when trucks are hoisted by cranes. XGBoost models have been widely adopted in the industry due to their exceptional reliability, precision, and portability [22,23]. Additionally, the modest requirements of XGBoost in terms of hardware resources serve to mitigate the costs of deployment, which would facilitate the widespread adoption of the proposed approach [24,25]. When trucks are hoisted, anomalies in the forces applied to the suspension systems arise, thereby inducing fluctuations in load weight. Furthermore, these fluctuations cause abnormal changes in both voltage and current within the drive motors of cranes. To identify these subtle anomalies, a mathematical model was established for drive motors [26,27] and suspension systems [28,29]. Employing captured electrical signals, the model calculates various physical parameters pertaining to the operational statuses of cranes. Subsequently, these derived physical parameters serve as training sets for the XGBoost model.

In the course of training the XGBoost model, the mathematical model augments datasets with more comprehensive representations of the physical information and selectively filters training sets in accordance with distinct hoists. We conducted experiments to verify that the filtered datasets markedly augment the performance of the XGBoost model. In the detection of truck hoisting, an initial analysis of input data is performed using the mathematical model, after which the filtered data are subjected to further assessment using the XGBoost model. The combined model demonstrates a substantial enhancement in performance relative to the individual XGBoost model.

Compared to other existing methods for truck hoisting detection, the advantages of the approach in this paper are reflected in the following aspects:

1. The proposed approach collects the electrical signals of motors using Hall sensors, and all devices utilized in the proposed approach can be deployed within the electrical control zones of cranes, ensuring the normal operation of the approach even in extreme weather conditions;
2. Hall sensors are more cost effective compared to both cameras and laser scanners, and can work stably for extended periods without calibration or frequent manual maintenance, contributing to relatively low operational times and costs;
3. The proposed approach can detect continuously during the spreader hoisting process without requiring containers to stop in mid-air, thereby improving unloading efficiency;
4. By constructing training sets using the mathematical model and combining that model with the XGBoost model, the proposed approach has high detection accuracy and low time complexity and responds quickly to abnormal situations.

## 2. Mathematical Model of Hoist Process

Driven by induction motors, spreaders elevate targeted containers for unloading. Through interactions between containers and trucks, suspension systems affect the elevation process upon contact. To characterize the ascent dynamics of trucks, a mathematical model for drive motors and suspension systems was built. The model inputs consist of voltage and current signals applied to drive motors, acquired using Hall sensors, which are subsequently transmitted for processing by microcomputers. The motor model enables the calculation of physical quantities that Hall sensors cannot measure directly, thereby enhancing the acquired physical information.

**Figure 1:**
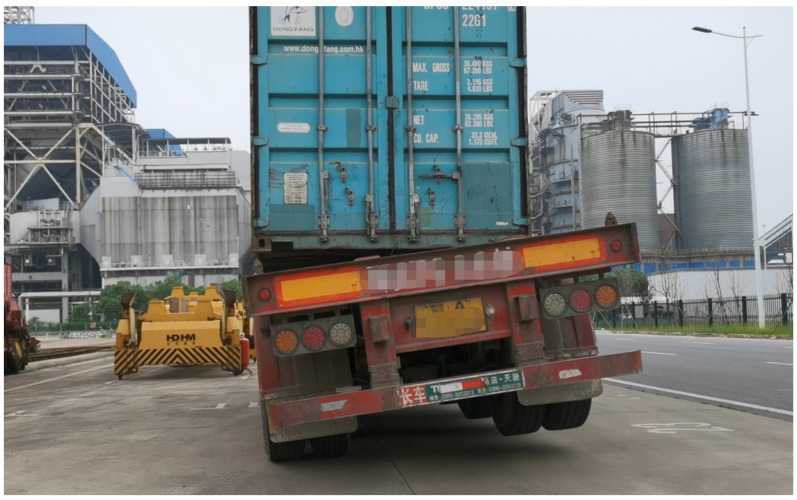
A truck being hoisted.

As illustrated in Figure 1, the locks between the truck and the container are incompletely disengaged. Consequently, during the unloading of the container, the underlying chassis is hoisted, causing an untoward incident.

### 2.1. Drive Motor Model and Parameters

**Figure 2:**
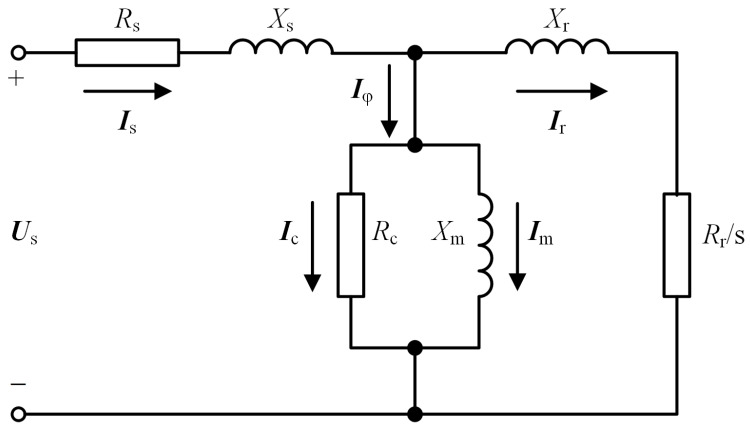
Equivalent circuit model of an induction motor.

An equivalent circuit model of an induction motor [30] is employed in this paper, as shown in Figure 2, where $U_{s}$ denotes stator line-to-neutral terminal voltage, $I_{s}$ is stator current, $I_{\varphi }$ represents exciting current, $I_{r}$ is the rotor current, and $I_{m}$ and $I_{c}$ are the magnetizing and core-loss components of $I_{\varphi }$, respectively.

The model presented in Figure 2 represents a single-phase equivalent circuit model of an induction motor, using parameters set to fixed values. In actual systems, the motor parameters are not constant due to factors such as temperature and humidity. However, these variations only have slight impacts on our study and can be disregarded. Additionally, stators and rotors are not electrically connected in actual systems, but are instead influenced by electromagnetic fields. To facilitate analysis, the model depicted in Figure 2 equates the relationship between the stator and rotor to an electrical circuit.

The model parameters were estimated from nameplate data and performance characteristics, including stator resistance $R_{s}$, rotor resistance $R_{r}$, core-loss resistance $R_{c}$, stator inductance $L_{s}$, rotor inductance $L_{r}$, and excitation inductance $L_{m}$. In Figure 2, $X_{s}$ and $X_{r}$ correspond to the leakage reactance associated with $L_{s}$ and $L_{r}$, respectively, while $X_{m}$ denotes the excitation reactance of $L_{m}$. Through the utilization of the motor model, additional physical information can be obtained, expanding the dimensions of input features in detection processes.

For drive motors employed in large-scale cranes, $R_{s}$ is relatively small and is conventionally treated as a constant. Within the confines of this paper, it is assumed that $X_{r}$ equates to $X_{s}$. The computed outcomes of the model parameters are listed in Table 1, with all parameters normalized.

### 2.2. Calculation of Physical Characteristics

In the preliminary stage, the root mean square (RMS) values of voltage V, current I, active power P, and frequency *f* are calculated based on the transient values gathered by Hall sensors. Subsequently, by incorporating the above-mentioned information into the motor model, the output power $P_{out}$, electromagnetic torque $T_{L}$, fractional slip *s*, motor speed *n*, and height *h* can be calculated.

In order to calculate frequency *f*, three-phase currents are transformed into $I_{\alpha }$ and $I_{\beta }$ using Clark conversion. The vector sum of $I_{\alpha }$ and $I_{\beta }$ forms an angle θ with the α-axis. The rotational angular velocity of the sum vector is equal to the synchronous angular velocity $\omega_{s}$ and can be calculated using Equation (1).

(1) $$\omega_{s}=\frac{d\theta }{dt}=\frac{d}{dt}\arctan\left(\frac{i_{\beta }}{i_{\alpha }}\right)=\frac{i_{\alpha }\frac{di_{\beta }}{dt}-i_{\beta }\frac{di_{\alpha }}{dt}}{i_{\alpha }^{2}+i_{\beta }^{2}}$$

The differential component within Equation (1) introduces a notable degree of noise. Subsequent to calculation, a depolarized mean filtration process is imperative to eliminate obvious pulse interference, thereby making the calculation result closer to the actual value.

In the model depicted in Figure 2, the input voltage is denoted as $e_{s}$, which is calculated by subtracting the voltage drop across $R_{s}$ from $U_{s}$. Applying Thevenin’s theorem on the stator side, the port-equivalent voltage is $V_{th}=\frac{X_{m}}{X_{s}+X_{m}}e_{s}$, alongside the equivalent reactance of $X_{th}=\frac{X_{s}X_{m}}{X_{s}+X_{m}}$. Given the motor model parameters, wherein $X_{s}$ markedly dwarfs $X_{m}$, approximations render $V_{th}$ nearly equivalent to $e_{s}$ and $X_{th}$ approximately equal to $X_{s}$.

The electromagnetic torque $T_{L}$ can be calculated by

(2) $$T_{L}=\frac{P_{gap}}{\omega_{s}}=\frac{qV_{th}R_{r}/s}{\omega_{s}\left[{\left(R_{r}/s\right)}^{2}+{\left(X_{th}+X_{r}\right)}^{2}\right]}$$

where *q* is the number of stator phases, *s* is the fractional slip, $P_{gap}=P_{in}-I^{2}R_{s}-e_{s}^{2}/R_{c}$ represents the air-gap power, and $P_{in}$ denotes the value of input active power.

Equation (2) can be transformed into a quadratic expression in terms of *s*, as illustrated below.

(3) $$P_{gap}{\left(X_{th}+X_{r}\right)}^{2}s^{2}-qV_{th}R_{r}s+P_{gap}R_{r}^{2}=0$$

In accordance with the parameters of the motor model, it can be deduced that the coefficient associated with the quadratic term in (3) is significantly smaller than those of the linear and constant terms. Consequently, the quadratic terms within (3) can be omitted. The computations for output power and motor speed can be calculated using $P_{out}=P_{gap}\left(1-s\right)$ and $n=\frac{60\omega_{s}}{2\pi p}\left(1-s\right)$, respectively.

The linear ascending velocity can be determined precisely via the application of coefficient $c_{nv}$, which delineates the relationship between the motor speed and operational velocity. Consequently, this facilitates the computation of the relative elevation *h* of containers.

### 2.3. Weight Calculation Algorithm

The proposed approach calculates the load weight of cranes using steady-state electrical parameters, which was introduced in [31] (crane load weight detection method, CLWD). The functional relationship established by CLWD can be described as

(4) $$m=f_{m}\left(P_{out},n\right)$$

According to the mapping $f_{m}$, the load weight *m* can be calculated using the output power $P_{out}$ and motor speed *n*.

### 2.4. Truck Suspension Model

**Figure 3:**
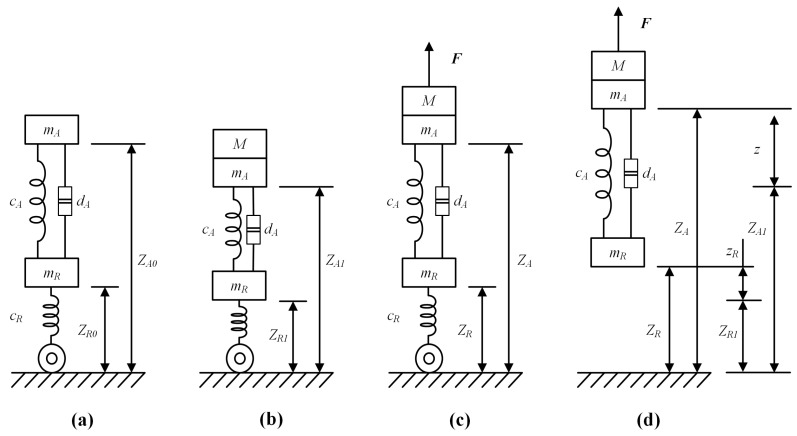
Simplified models of truck suspension in four states: (**a**) a motionless truck without a container; (**b**) a motionless truck with a container; (**c**) a spreader hoisting a container; (**d**) a truck being hoisted.

Suspension systems comprise various components, such as guiding mechanisms, elastic elements, trapezoidal structures, damping elements, stabilizing devices, and limiting rubber blocks. In the context of truck hoisting detection, the focus is on the longitudinal displacement. Consequently, a suspension system model was developed, considering elastic elements, damping elements, and wheels. Suspension is simplified into a spring-damping system, as depicted in Figure 3, where $d_{A}$ denotes the stiffness coefficient of the truck damper, $c_{A}$ represents the stiffness of the truck’s steel plate spring, $c_{R}$ is the stiffness of the tires, $Z_{R0}$, $Z_{R1}$, and $Z_{R}$ are the heights of the tire center from the ground, $Z_{A0}$, $Z_{A1}$, and $Z_{A}$ are the heights of the chassis plane from the ground, and $m_{R}$, $m_{A}$, and *M* represent the weights of the tires, truck, and container, respectively.

The parameters of the suspension model utilized in this paper are presented in Table 2, with normalization applied to the parameters.

Considering a container that is pulled upward by a spreader with a force of F, the simplified suspension model can be described as follows:

(5) $$\left\{\begin{array}{c} \left(M+m_{A}\right)\ddot{z}=F-c_{A}\left(z-z_{R}\right)-d_{A}\left(\dot{z}-{\dot{z}}_{R}\right)\\ m_{R}{\ddot{z}}_{R}=c_{A}\left(z-z_{R}\right)+d_{A}\left(\dot{z}-{\dot{z}}_{R}\right)-c_{R}z_{R} \end{array}\right.$$

where *z* is the height of the container, $z_{R}$ is the displacement of the tire center, and $\dot{z}$ and $\ddot{z}$ denote the first and second derivatives of *z*, respectively.

Moreover, the relationship between *z* and $z_{R}$ can be represented as in Equation (6) if the truck is off the ground.

(6) $$m_{R}{\ddot{z}}_{R}=c_{A}\left(z-z_{R}\right)+d_{A}\left(\dot{z}-{\dot{z}}_{R}\right)-\left(M+m_{A}+m_{R}\right)g$$

where *g* is the gravitational acceleration, which has a value of 9.8 m/s^2^ in this study.

**Figure 4:**
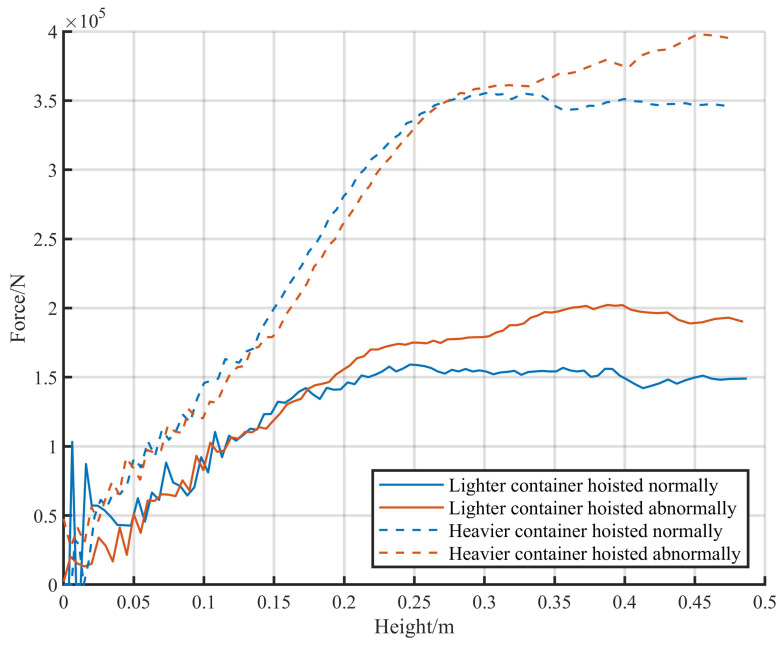
Calculation results for different cases.

Physical characteristics change in different statuses of hoists. By analyzing these distinctions, the range of physical quantities under different statuses can be constrained. In this paper, hoists are divided into two statuses: normal and abnormal. The experimental data for container hoists in different statuses were collected separately. The variations in tension *F* with height *h* under different statuses can be calculated using (5) and (6). Let z=h and M=m, where *M* includes the weight of the spreader. The calculation results are shown in Figure 4.

The calculation results indicate that the heavier the container, the higher the corresponding height when the pulling force is stable. During abnormal hoists, the suspension spring undergoes reverse deformation, leading to different characteristics compared to normal hoists. The corresponding height in abnormal hoists is higher than that of normal hoists.

Based on the characteristics of unloading, the suspension model imposes physical restrictions on normal hoists, such as weight and height. On the other hand, abnormal situations can be identified by the suspension model based on the characteristics of calculated load weight and other physical quantities. However, due to the complexity of on-site conditions, similarities in many features between normal and abnormal hoists can result in false positives. To improve the accuracy of detection, a more precise XGBoost model [32] is employed due to its high reliability, low computational time complexity, and high accuracy [33].

## 3. Detection Algorithm for Abnormal Hoists

**Figure 5:**
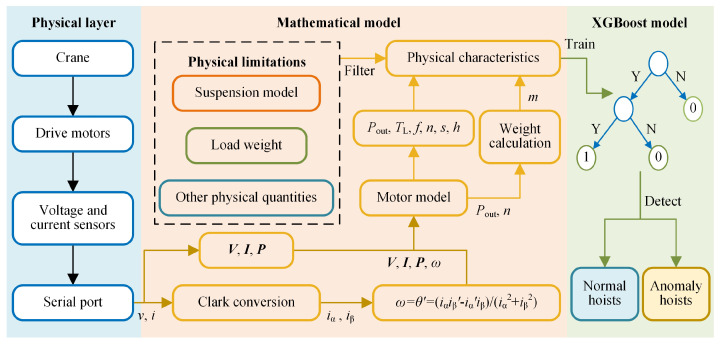
Structure of the algorithm proposed in this paper.

In this paper, cranes are driven by induction motors. The voltage and current signals of these motors are gathered using Hall sensors and transmitted via serial ports to microcomputers. Their physical characteristics are calculated by the motor model and the weight calculation algorithm. Furthermore, the characteristics are then filtered by physical limitations using the suspension model, load weight, and other physical quantities. To identify abnormal hoists, the XGBoost model is trained using the filtered characteristics. The structure of the proposed algorithm is depicted in Figure 5. The proposed approach recognizes abnormal hoists by using both the mathematical model and the XGBoost model. For hoists identified as normal by the mathematical model, the XGBoost model ceases further computations. Alerts are sent to the control systems of cranes and unloading operations are shut down in a timely manner when exceptions are identified. During detection, the mathematical model processes datasets and improves the physical information, thereby enhancing the training datasets for the XGBoost model.

### 3.1. Events and Samples

**Figure 6:**
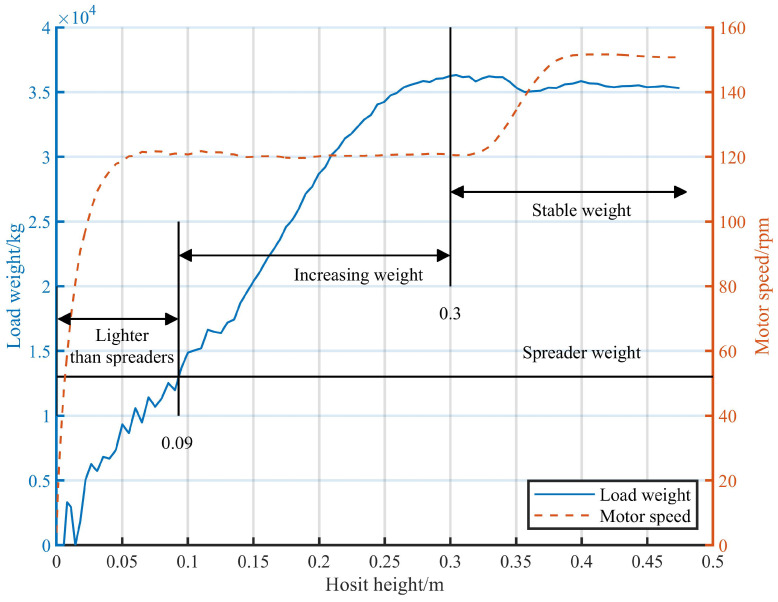
Schematic diagram of the three hoisting stages.

The continuous monitoring of the electrical signals in the motors is conducted via Hall sensors. Since signals only contain slight noise during the idle state of cranes, hoists can be discernible by analyzing variations in voltage and current. In order to improve the reliability of detection results, this study carries out periodic detections at 0.05-s intervals during the entire hoisting process. Detections persist until the given termination conditions have been satisfied. For the identification of initiation and conclusion timings, a single hoist can be divided into three stages, as shown in Figure 6.

The three hoisting stages can be defined as follows:

- Lighter than spreaders: A wire rope is straightened gradually, during which the load weight *m* is less than the spreader weight, which occurs within the range of the *x*-axis, from 0 to 0.09, as depicted in Figure 6;
- Increasing weight: The container ascends slowly until lifted off the ground or the truck completely, throughout which the load weight *m* gradually increases within the range of the *x*-axis, from 0.09 to 0.3, as shown in Figure 6;
- Stable weight: Entire load is off the ground and *m* is relatively stable within the range beyond 0.3 on the *x*-axis, as shown in Figure 6.

It can be inferred that the hoisting statuses of trucks are indicated during the increasing weight stage, in which detection data are gathered. Each detection collects 3 s of data and terminates upon the satisfaction of the given load weight conditions. In this paper, the term “sample” denotes the data utilized in each individual detection, whereas an “event” is defined as the aggregate of samples from a single hoisting instance. In addition, positive and negative denote normal and abnormal events, respectively.

Detection samples are represented by matrices $D_{t}\in R^{60\times 16}$ in this paper. These matrices are the fundamental units for detection analysis and consist of physical time series data (V, I, P, $P_{out}$, $T_{L}$, *f*, *n*, *s*, *h*, and *m*). To standardize the length of detection data, a sliding window method is employed to gather the sample data.

### 3.2. Mathematical Model Detection

The mathematical model extends the physical information of raw data and improves the training datasets for the XGBoost model. Moreover, the mathematical model provides a coefficient matrix $A\in R^{c\times d}$ and a detection threshold vector $\theta ={\left[\begin{array}{cccc} \theta_{1} & \theta_{2} & \cdots & \theta_{c} \end{array}\right]}^{T}$ for detection, where *c* is the number of constraints and *d* is the length of the input vector.

For a vector $K_{m,t}$ extracted from a sample $D_{t}$, the detection result given by the mathematical model is

(7) $$y_{m}\left(t\right)=\frac{1}{c}\sum_{j=1}^{c}f_{u}\left(x{\left(t\right)}^{\left(j\right)}\right)$$

where $x\left(t\right)={\mathrm{AK}}_{m,t}-\theta$, $K_{m,t}=\left[\begin{array}{c} E_{t}\\ F_{t}\\ G_{t} \end{array}\right]$, $f_{u}{(x\left(t\right)}^{\left(j)\right)}=\left\{\begin{array}{c} 0,\;if\;x{\left(t\right)}^{\left(j\right)}<0\\ 1,\;if\;x{\left(t\right)}^{\left(j\right)}\geq 0 \end{array}\right.$, $x{\left(t\right)}^{\left(j\right)}$ denotes the *i*th element of vector x(t), and $x{\left(t\right)}^{\left(j\right)}<0$ indicates the satisfaction of the *j*th condition, $E_{t}=\sum_{k=0}^{L-1}G\left(t\right)$, $F_{t}=G\left(t\right)-G\left(t-L+1\right)$, $G\left(t\right)={\left[\begin{array}{ccccccc} P_{out}\left(t\right) & T_{L}\left(t\right) & f(t) & n(t) & s(t) & h(t) & m(t) \end{array}\right]}^{T}$, and L=60.

To minimize false positives (FPs) and false negatives (FNs), outcome averaging is employed across multiple samples to obtain the detection result of an event. Additionally, a relatively strict threshold $\theta_{m}$ is set to prevent FNs as much as possible. The overall abnormality degree for an event can be represented by $Y_{m}=\frac{1}{N}\sum_{k=0}^{N-1}y_{m}\left(t-k\right)$ in the mathematical model, where *N* is the number of samples in an event.

Due to the difficulty in collecting abnormal samples, a significant imbalance can be observed between normal and abnormal samples. Moreover, the quality of normal samples varies widely. The primary function of the mathematical model lies in the exclusion of low-quality normal samples from training sets and preliminary detections during runtime.

### 3.3. Mathematically Enhanced XGBoost Algorithm for Truck Hoisting Detection

**Figure 7:**
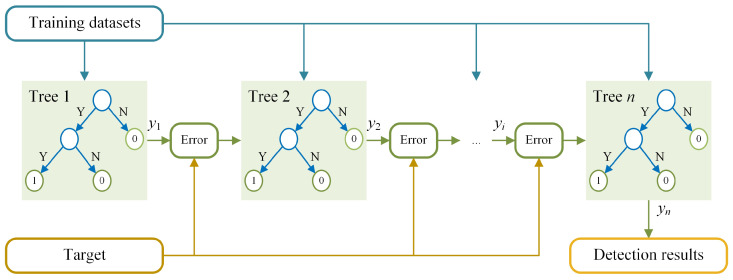
Structure of XGBoost model.

The mathematical model can identify most normal events, while for complex examples, it can yield false results. In order to reduce FPs and FNs, the XGBoost model is trained based on improved datasets. In this paper, Optuna [34,35] is used to run hyperparameter optimization and search for the optimal hyperparameters of the XGBoost model via cross-validation. The XGBoost model consists of a series of CART decision trees, as shown in Figure 7.

Utilizing training datasets $D=\{D_{t}|y_{m}\geq \theta_{m}\}$, new trees are generated based on errors between previous results $y_{i}$ and the target. Furthermore, the XGBoost model aggregates the results of each tree to obtain an overall prediction.

For a given input vector $K_{X,t}$, the detection result produced by XGBoost can be described by

(8) $$y_{X}\left(t\right)=\sum_{j}T_{j}\left(K_{X,t},\Theta_{j}\right)$$

where $T_{j}\left(K_{X,t},\Theta_{j}\right)$ represents the *j*th decision tree and $\Theta_{j}$ denotes the parameters of the decision tree.

Similar to the results of mathematical model, the XGBoost model uses the average abnormality degree $Y_{X}=\frac{1}{N}\sum_{k=0}^{N-1}y_{X}\left(t-k\right)$ and abnormality threshold $\theta_{X}$ for the entire hoisting event.

Since the training datasets for the XGBoost model are constructed using the mathematical model, the detection order of the two models cannot be swapped during actual testing. The detection result for a hoisting event can be expressed as

(9) $$Y=f_{u}\left(\left(Y_{m}-\theta_{m}\right)\left(Y_{X}-\theta_{X}\right)\right)$$

## 4. Experiments

**Figure 8:**
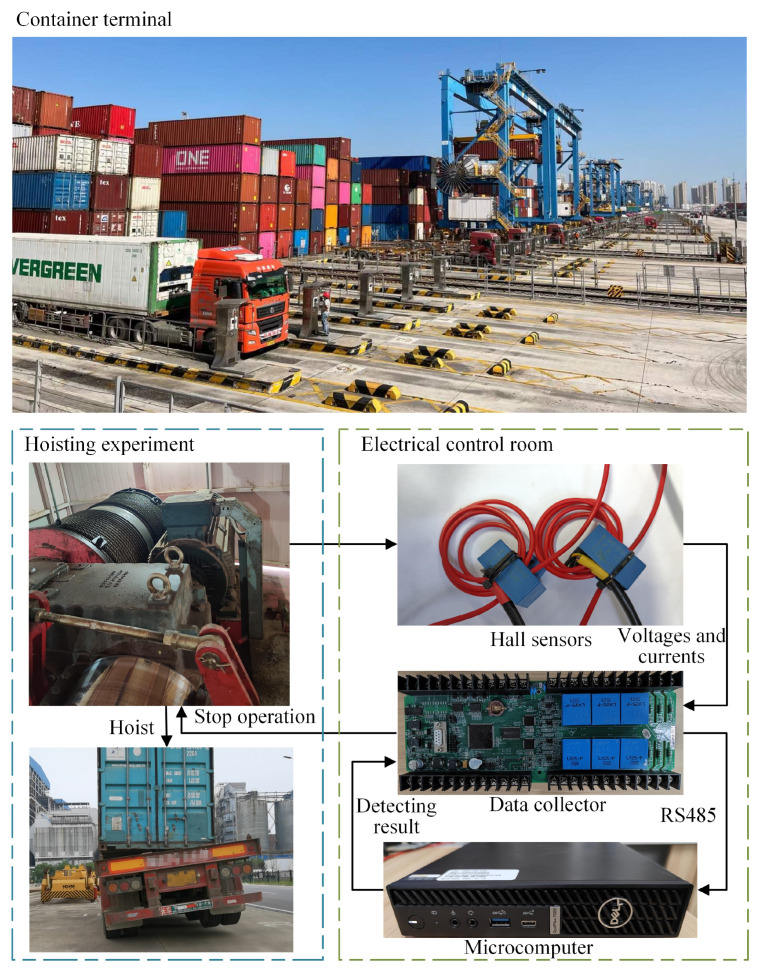
Schematic diagram of the field experiments.

The detection algorithm was deployed in a number of container terminals to verify its accuracy through experiments. A schematic diagram of the field experiments is shown in Figure 8.

### 4.1. Assessment Metrics

The false positive rate (FPR) and false negative rate (FNR) of all events were used as direct indicators for evaluating model effectiveness. FPR represents the probability of incorrectly predicting a sample or an event as positive among all negative instances. FPR and FNR can be calculated using $FPR=\frac{FP}{FP+TN}$ and $FNR=\frac{FN}{TP+FN}$, where true negatives (TNs) denote correctly predicted negative instances and true positives (TPs) are correctly predicted positive instances.

Considering that an event comprises multiple samples, the FPRs and FNRs for samples were also calculated. Due to the substantial imbalance between normal and abnormal datasets in this study, both the FPRs and FNRs were significantly influenced by the chosen threshold. Consequently, this study employed the area under the receiver operating characteristic curve (AUROC) [36,37] as it is an evaluative measure that is unaffected by threshold values.

In a receiver operating characteristic (ROC) curve [38,39], the horizontal and vertical axes represent FPR and true positive rate (TPR) at different thresholds, respectively, where FPR and TPR pertain to samples. TPR can be calculated using $TPR=\frac{TP}{TP+FN}$.

### 4.2. Collection of Datasets

Raw data were collected via the monitoring of standard operations and truck hoisting experiments under different conditions. Since numerous factors can affect the characteristics of operations, this study obtained more comprehensive datasets by altering containers, hoisting speeds, and twist locks.

**Figure 9:**
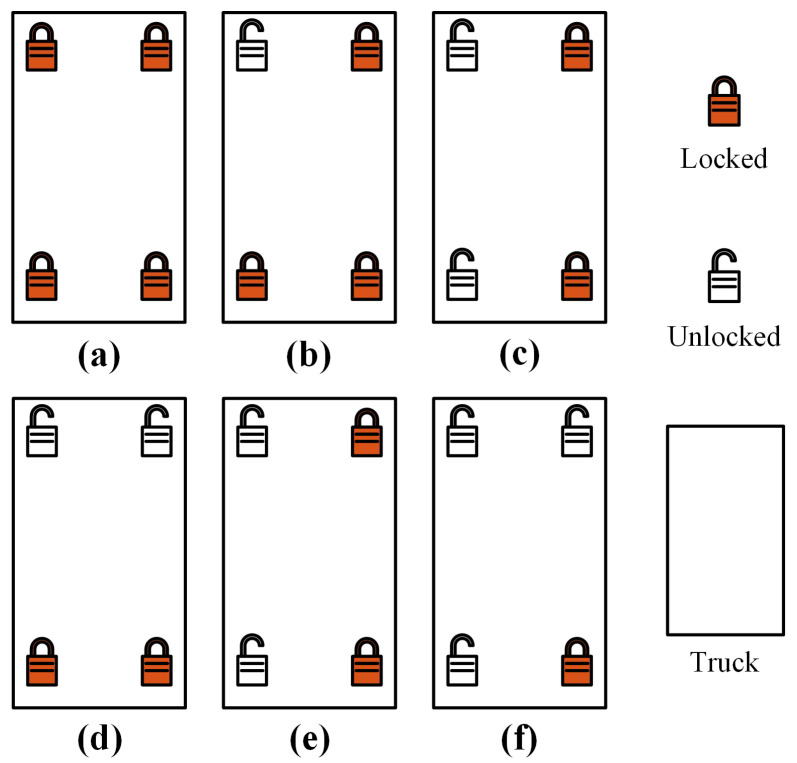
Top-down views of the six cases of twist locks: (**a**) all four twist locks are locked; (**b**) three twist locks are locked; (**c**) two diagonal twist locks are locked; (**d**) two twist locks on the short side of the container are locked; (**e**) two twist locks on the long side of the container are locked; (**f**) one twist lock is locked.

Among the three considered factors, the differences in twist locks could be divided into six cases, as shown in Figure 9.

This study conducted a series of experiments by modifying containers, hoisting speeds, and twist locks. The detailed experimental procedures was as follows:

1. Equipment preparation: The experimental equipment encompassed a truck and a container. It was imperative to ensure that the twist locks were functional and have replacement locks readily available. Subsequent to the preparation of the experimental equipment, a crane operator positioned a container on a truck smoothly and awaited the ground operator to fasten the twist locks.
2. Hoisting simulation: The crane operator hoisted the container from the truck, ensuring that the hoisting time exceeded 3 s while maintaining safety precautions. The detection system activated an alarm and restricted the ascent of the spreader when anomalies were identified. Subsequently, the crane operator lowered the container slowly in response to the alarm. When the truck was about to exceed the maximum safe height without triggering an alarm, the lifting operation was promptly terminated.
3. Experiment repetition: Steps 1 and 2 were repeated, varying the twist locks and hoisting speeds.

Following the completion of experiments on one current container, it was replaced with another container and the aforementioned steps were repeated.

### 4.3. Ablation Experiments

This study conducted six experiments using data from the Wuhan region. Improvements in the mathematical model were assessed by comparing the AUROCs of different models. Experiments 1, 2, and 3 were conducted without applying the mathematical model in the detection process, while experiments 4, 5, and 6 employed the mathematical model. The detailed results for each experiment are presented in Table 3.

Experiments 1, 2, and 3 demonstrated significant improvements in model performance through the utilization of the PQ training datasets. This conclusion was further reinforced by the comparative analysis of experiments 5 and 6 against experiment 4, where the former models demonstrated superior efficacy. Both sets of experiments underscored that the employment of the mathematical model could highlight data characteristics effectively. This enhancement facilitated the differentiation between normal and abnormal samples, resulting in models with improved performance.

Datasets are characterized by a significant imbalance between normal and abnormal events in this paper. The positive cooperation of both port personnel and truck drivers was essential for the collection of experimental data. The elevated hazard factor further intensified the challenges associated with collecting a substantial volume of abnormal data. Moreover, the instances of unloading containers from container trucks were relatively scarce within normal events, resulting in an uneven distribution of normal training data. The initial normal datasets comprised operations involving the unloading of containers from trucks, hoisting containers from the yard, and the hoisting of spreaders. The low quality of these datasets constrained the performance of the models.

**Figure 10:**
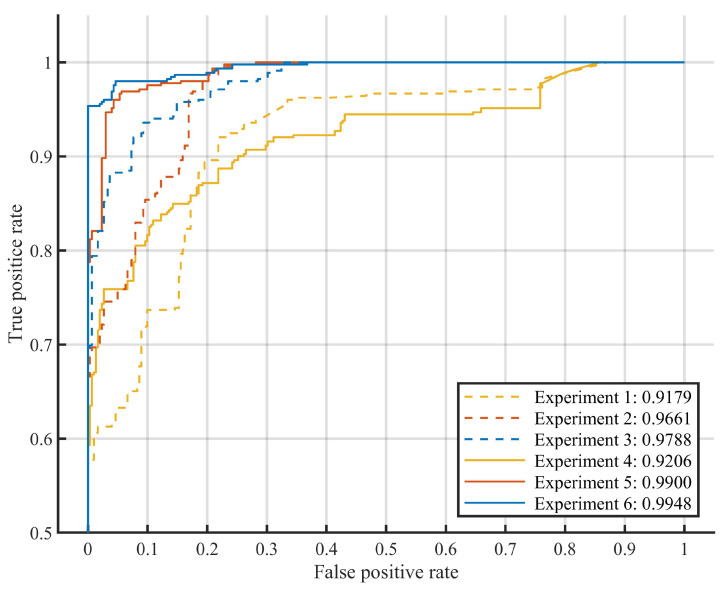
ROC curves of the models.

In comparison to the models employed in experiments 1, 2, and 3, each model in experiments 4, 5, and 6 demonstrated distinct improvements in performance. The models employed in experiments 4, 5, and 6 utilized datasets filtered by the mathematical model, with preliminary predictions applied to samples during the detection process. Conversely, the models for experiments 1, 2, and 3 utilized datasets without prior filtering. The mathematical model could effectively address the issues of data imbalance and low quality by filtering the normal datasets. The quality of the filtered datasets was enhanced, better reflecting the distinction between normal and abnormal events, consequently leading to improved model performance. This enhancement is illustrated in the ROC curves presented in Figure 10. The ROC curves in Figure 10 illustrate the relationships between the true positive rates and false positive rates in the six experiments. The false positive rate is related to the setting of an abnormality threshold, where a higher abnormality threshold leads to a lower false positive rate. However, a larger abnormality threshold also results in a lower true positive rate, indicating a higher false negative rate. The ROC curve for experiment 6 reaches above the other curves over much of the *x*-axis range, which indicates that the model in experiment 6 had better results across a wide range of abnormality thresholds.

### 4.4. Assessments

The proposed approach in this study was implemented on cranes across several cities (Ningbo, Qingdao, Wuhan, and Qinzhou) to continuously monitor the routine operations of cranes. Across these cities, the variables under measurement included containers, hoisting speeds, and the positions and quantities of twist locks. The same truck was employed in each city for abnormal data collection and various other data were collected by altering the measured variables. During the collection of normal data, no interventions were made to the cranes, allowing them to maintain regular operation. Detailed information about the cranes and motors was collected at each station to calculate the corresponding parameters for the mathematical model. A set of data was collected for the purpose of training the XGBoost model. In addition, adjustments were made to parameters like hoisting speed and the weight of the spreader, ensuring the adaptability of the proposed approach for various stations.

Throughout the monitoring period, no incidents of truck hoisting occurred. Therefore, we conducted experiments on trucks hoisting following the steps detailed in Section 4.1 to evaluate the FNRs of the models across different regions. The results of these regional experiments are presented in Table 4 and Table 5.

Regarding the detection of events, the approach demonstrated an FPR below 0.7% across all regions and a zero FNR, as shown in Table 4. While sample detection encountered occasional false negatives, as shown in Table 5, the aggregate processing of multiple samples effectively mitigated the oversight of abnormal events. In order to decrease false negative instances, further analysis will be conducted on the false negative samples and greater weight will be assigned to them in subsequent training phases. Consequently, the approach ensures consistently low FPRs in the routine operations of cranes while being responsive to the hoisting of trucks, thus offering a protective measure for port security.

## 5. Conclusions

This paper introduced a detection algorithm for the hoisting of trucks based on electrical signals. The approach utilizes sensors to collect input current and voltage data from drive motors, without relying on visual, rotational speed, position, tension, or pressure sensors. A mathematical model is employed to compute operational state information during the hoisting process. By expanding the data dimensions, additional pertinent physical information is extracted, thereby reducing the time costs of information acquisition. The approach combines this mathematical model with an XGBoost model, demonstrating low computational complexity and improved accuracy.

Microcomputers and data collectors were installed on several cranes in this study. The algorithm was deployed on microcomputers and the effectiveness and accuracy of the proposed method were experimentally validated. The experimental results demonstrated that the detection algorithm in this study had no false negative events. Furthermore, the detection algorithm exhibited an overall false positive rate of below 1% over a continuous operational period. The algorithm sends an alarm signal to the crane control system to stop the hoisting operation when an anomaly is detected. The identification and alarm processes operate expeditiously, thereby ensuring the height of the truck stays within a safe threshold.

In summary, the proposed approach employs maintenance-free sensors and possesses multiple advantages, including convenient deployment, negligible interference from device operation, computational simplicity, and high accuracy. Moreover, the approach could be employed to ensure the safety and loading efficiency of hoisting processes, which holds significant economic and social value. Future research will focus on how to improve the portability of the proposed approach, so that the approach can be deployed and used directly in new stations with a few or even no experiments. Considering the distinctions between the drive motors of different cranes, we intend to map these motors to the same standard based on the rated parameters of the motors. As accuracy requirements continue to increase, we will also use different noise reduction methods and more accurate sensors. In addition, accuracy needs to be further improved and we hope to explore the applicability of the proposed method in different scenarios, such as unloading containers from trains, ships, etc.

## Figures and Tables

**Table 1:** Normalized parameters of the motor model.

Parameter	Normalized Value
$R_{s}$	2.84 × 10^−4^
$R_{r}$	3.06 × 10^−3^
$R_{c}$	1
$L_{s}$	1.39 × 10^−5^
$L_{r}$	1.39 × 10^−5^
$L_{m}$	1.28 × 10^−3^

**Table 2:** Normalized parameters of the suspension model.

Parameter	Normalized Value
$m_{A}$	6.83 × 10^−2^
$m_{R}$	1.02 × 10^−3^
$d_{A}$	1.21 × 10^−2^
$c_{A}$	1.21 × 10^−1^
$c_{R}$	1

**Table 3:** Results of the ablation experiments.

Experiment	Training Datasets		Models		AUROC
	EQ ^1^	PQ ^2^	MM ^3^	XGBoost	
1	✓			✓	0.9179
2	✓	✓		✓	0.9661
3		✓		✓	0.9788
4	✓		✓	✓	0.9206
5	✓	✓	✓	✓	0.9900
6		✓	✓	✓	0.9948

^1^ ES, electrical quantities (V, I, and P); ^2^ PQ, physical quantities calculated by the mathematical model ($P_{out}$, $T_{L}$, *f*, *n*, *s*, *h*, and *m*); ^3^ MM, mathematical model.

**Table 4:** Results of regional assessments for events.

Crane Identification Code	Normal Events	Abnormal Events	FPR	FNR
Ningbo 01	4907	55	0.02%	0.00%
Ningbo 02	3776	52	0.05%	0.00%
Ningbo 08	5391	149	0.04%	0.00%
Qingdao	17,922	223	0.44%	0.00%
Wuhan	8177	298	0.48%	0.00%
Qinzhou 01	16,312	165	0.69%	0.00%
Qinzhou 18	6053	356	0.53%	0.00%

**Table 5:** Results of regional assessments for samples.

Crane Identification Code	Normal Samples	Abnormal Samples	FPR	FNR
Ningbo 01	65,427	473	0.02%	2.54%
Ningbo 02	18,909	302	0.00%	0.02%
Ningbo 08	41,310	1871	0.03%	0.05%
Qingdao	258,175	2594	0.41%	0.15%
Wuhan	52,127	3984	1.14%	0.53%
Qinzhou 01	122,105	2295	0.83%	0.35%
Qinzhou 18	48,944	5161	1.20%	0.54%

## Data Availability

Embargo on data due to commercial restrictions.

## Abbreviations

The following abbreviations are used in this manuscript:

XGBoost	Extreme gradient boosting
PSLS	Method utilizing photoelectric sensors and laser scanners
RMS	Root mean square
CLWD	Crane load weight detection method
TP	True positive
FP	False positive
TN	True negative
FN	False negative
FPR	False positive rate
FNR	False negative rate
TPR	True positive rate
ROC	Receiver operating characteristic
AUROC	Area under ROC curve
EQ	Electrical quantities (V, I, and P)
MM	Mathematical model
PQ	Physical quantities calculated by MM ($P_{out}$, $T_{L}$, *f*, *n*, *s*, *h*, and *m*)
